# A checklist and areography of longhorn beetles (Coleoptera: Cerambycidae) in Rila Mountain

**DOI:** 10.3897/BDJ.9.e72494

**Published:** 2021-10-18

**Authors:** Georgi Georgiev, Vladimir Sakalian, Plamen Mirchev, Margarita Georgieva, Sevdalin Belilov

**Affiliations:** 1 Forest Research Institute - Bulgarian Academy of Sciences, Sofia, Bulgaria Forest Research Institute - Bulgarian Academy of Sciences Sofia Bulgaria; 2 Institute of Biodiversity and Ecosystem Research - Bulgarian Academy of Sciences,, Sofia, Bulgaria Institute of Biodiversity and Ecosystem Research - Bulgarian Academy of Sciences, Sofia Bulgaria

**Keywords:** Cerambycidae, Rila Mt., areography, Bulgaria

## Abstract

**Background:**

The complex of longhorn beetles in Rila Mt. in Bulgaria was studied by literature data and original biological materials. As a result, 126 taxa from six subfamilies were established, as follows: Prioninae (four taxa), Lepturinae (43 taxa), Necydalinae (two taxa), Spondylidinae (seven taxa), Cerambycinae (31 taxa) and Lamiinae (39 taxa).

**New information:**

In this study, two new records for Rila Mt. (*Stenurellanigranigra* and *Xylosteusspinolae*) and new localities or additional information for 24 cerambycid taxa were reported. The longhorn beetles belong to 18 zoogeographical categories and seven complexes. The European complex occupies a dominant position (37.3%), followed by the Palaearctic (23.8%), Eurosiberian (13.5%), Mediterranean (11.1%), European-Iranoturanian (7.1%), Balkan endemic (4.0%) and Holarctic (3.2%) complexes.

## Introduction

Rila is the highest and one of the largest mountains in Bulgaria. The average altitude is 1487 m a.s.l. and the total area - 2629 km^2^. The highest peak of the mountain, Musala (2925 m a.s.l.), is the highest on the Balkan Peninsula and in Eastern Europe (Ivanov 1966).

In Rila Mt., there is a large number of tree and shrub species clearly distributed in vegetation belts. The deciduous belt is formed mainly by hornbeam (*Carpinusbetulus* L.), sessile oak (*Quercuspetraea* (Matt.) Liebl.), common beech (*Fagussylvatica* L.), aspen (*Populustremula* L.) and birch (*Betulapendula* Roth) and coniferous one - by silver fir (*Abiesalba* Mill.), Norway spruce (*Piceaabies* (L.) Karst.), Scots pine (*Pinussylvestris* L.), Balkan pine (*Pinuspeuce* Griseb.) and dwarf mountain pine (*Pinusmugo* Turra) (Stoyanov 1966).

Information about findings of longhorn beetles of Rila is available in a number of literature sources (Heyrovský 1931, Kantardjiewa-Minkova 1932, Kantardjiewa-Minkova 1934, Minkova 1957, Minkova 1961, Angelov 1967, Angelov 1995, Ganev 1984, Ganev 1985, Ganev 1986, Doychev and Georgiev 2004, Migliaccio et al. 2007, Rapuzzi and Georgiev 2007 etc.). However, there is no check-list of cerambycid fauna of the mountain.

The aim of this study is to sumarise data in entomological literature about longhorn beetles in Rila Mt., to report new records of longhorn beetles and to make zoogeographical analysis of cerambycid fauna in the mountain.

## Materials and methods

The longhorn beetles of Rila Mt. were studied by literature data, original records and unpublished materials in entomological collections. The original material was collected on flowers and host plants.

In this study, classification and nomenclature of the longhorn beetles suggested by Sama 2002, Sama 2013, Téocchi 2003, Biscaccianti 2007, Lobl and Smetana 2010, Özdikmen 2011, Miroshnikov 2016 and Danilevsky 2021 are followed, without indication of tribes and subgenera. Some taxa reported from Rila Mt., but most likely misidentified, are not included in the list: *Agapanthialais* Reiche & Saulcy, 1858 (Bringmann 1995) and *Phytoeciaaffinisnigropubescens* G. Müller, 1948 (reported as *Phytoecianigripesnigropubescens* in Bringmann (1998)).

The zoogeographical characterisation of longhorn beetles was made on the basis of recent taxa distribution (Danilevsky 2021). According to Georgiev and Hubenov (2006) and Sakalian and Langourov (2007) conceptions, the established taxa are arranged in 18 chorotypes (areographic categories).

The new cerambycid records are deposited in entomological collection of Georgi Georgiev (mentioned with the abbreviation [GG]).

## Checklists

### Checklist

#### Prionus coriarius

(Linnaeus, 1758)

4D23706C-5FFF-50E6-94C2-0EA40A8EDA35

##### Materials

- **Type status:** Other material. **Occurrence:** recordedBy: V. Radkova leg. [GG]; sex: 2 males; **Location:** country: Bulgaria; locality: Brashantsi vill.; **Event:** eventDate: 13-08-81

##### Distribution

West Palaearctic species (Danilevsky 2021)

#### Alosterna tabacicolor tabacicolor

(DeGeer, 1775)

BB231D19-87F9-5068-A839-A14E4C975F8C

##### Materials

- **Type status:** Other material. **Occurrence:** recordedBy: G. Georgiev leg. [GG]; sex: 1 female; **Location:** country: Bulgaria; locality: Parangalitsa; verbatimElevation: 1300 m a.s.l.; **Event:** eventDate: 07-20-04

##### Distribution

West Eurosiberian subspecies (Danilevsky 2021)

#### Anastrangalia dubia dubia

(Scopoli, 1763)

356627BD-7DA8-5437-88C5-874CE622DEDE

##### Materials

- **Type status:** Other material. **Occurrence:** recordedBy: G. Georgiev leg. [GG]; sex: 3 males, 2 females; **Location:** country: Bulgaria; locality: Parangalitsa; verbatimElevation: 1300 m a.s.l.; **Event:** eventDate: 07-20-04
- **Type status:** Other material. **Occurrence:** recordedBy: G. Georgiev leg. [GG]; sex: 1 male, 3 females; **Location:** country: Bulgaria; locality: Rila Monastery; verbatimElevation: 1400 m a.s.l.; **Event:** eventDate: 07-07-04
- **Type status:** Other material. **Occurrence:** recordedBy: G. Georgiev leg. [GG]; sex: 3 males, 1 female; **Location:** country: Bulgaria; locality: Treshtenik loc.; verbatimElevation: 1250 m a.s.l.; verbatimLatitude: 42.052222; verbatimLongitude: 23.668694

##### Distribution

Euromediterranean subspecies (Danilevsky 2021)

#### Anastrangalia sanguinolenta

(Linnaeus, 1760)

D1029338-707B-5B6C-A116-D6D8A055FFBB

##### Materials

- **Type status:** Other material. **Occurrence:** recordedBy: G. Georgiev leg. [GG]; sex: 4 males; **Location:** country: Bulgaria; locality: Parangalitsa; verbatimElevation: 1300 m a.s.l.; **Event:** eventDate: 07-20-04
- **Type status:** Other material. **Occurrence:** recordedBy: G. Georgiev leg. [GG]; sex: 1 male, 1 female; **Location:** country: Bulgaria; locality: Treshtenik loc.; verbatimElevation: 1250 m a.s.l.; verbatimLatitude: 42.052222; verbatimLongitude: 23.668694

##### Distribution

West Eurosiberian species (Danilevsky 2021)

#### Leptura quadrifasciata quadrifasciata

Linnaeus, 1758

7FBBA3D2-C3A6-5495-9F9D-8863C6D1ABDA

##### Materials

- **Type status:** Other material. **Occurrence:** recordedBy: G. Georgiev leg. [GG]; sex: 1 male; **Location:** country: Bulgaria; locality: Parangalitsa; verbatimElevation: 1300 m a.s.l.; **Event:** eventDate: 07-20-04

##### Distribution

Transpalaearctic subspecies (Danilevsky 2021)

#### Judolia cerambyciformis

(Schrank, 1781)

A70E73E3-C2AB-5F61-9090-8A81C6BFFFB1

##### Materials

- **Type status:** Other material. **Occurrence:** recordedBy: G. Georgiev leg. [GG]; sex: 1 male, 1 female; **Location:** country: Bulgaria; locality: Rila Monastery; verbatimElevation: 1400 m a.s.l.; **Event:** eventDate: 07-07-04
- **Type status:** Other material. **Occurrence:** recordedBy: G. Georgiev leg. [GG]; sex: 1 female; **Location:** country: Bulgaria; locality: Harsovo vill.; verbatimElevation: 800 m a.s.l.; **Event:** eventDate: 07-20-04
- **Type status:** Other material. **Occurrence:** recordedBy: G. Georgiev leg. [GG]; sex: 1 male; **Location:** country: Bulgaria; locality: Parangalitsa; verbatimElevation: 1300 m a.s.l; **Event:** eventDate: 07-20-04
- **Type status:** Other material. **Occurrence:** recordedBy: G. Georgiev leg. [GG]; sex: 1 male; **Location:** country: Bulgaria; locality: Ovtchartsi vill.; verbatimElevation: 900 m a.s.l.; **Event:** eventDate: 07-21-04
- **Type status:** Other material. **Occurrence:** recordedBy: G. Georgiev leg. [GG]; sex: 2 males, 1 female; **Location:** country: Bulgaria; locality: Treshtenik loc.; verbatimElevation: 1250 m a.s.l.; verbatimLatitude: 42.052222; verbatimLongitude: 23.668694

##### Distribution

European species (Danilevsky 2021)

#### Rutpela maculata maculata

(Poda von Neuhaus, 1761)

9B3A3AE8-721E-5BD2-9949-FEFB1B4A7B6A

##### Materials

- **Type status:** Other material. **Occurrence:** recordedBy: G. Georgiev leg. [GG]; sex: 1 female; **Location:** country: Bulgaria; locality: Rila Monastery; verbatimElevation: 1400 m a.s.l.; **Event:** eventDate: 07-07-04
- **Type status:** Other material. **Occurrence:** recordedBy: G. Georgiev leg. [GG]; sex: 1 male, 1 female; **Location:** country: Bulgaria; locality: Ovtchartsi vill.; verbatimElevation: 900 m a.s.l.; **Event:** eventDate: 07-21-04
- **Type status:** Other material. **Occurrence:** recordedBy: G. Georgiev leg. [GG]; sex: 2 males; **Location:** country: Bulgaria; locality: Treshtenik loc.; verbatimElevation: 1400 m a.s.l.; verbatimLatitude: 42.052222; verbatimLongitude: 23.668694

##### Distribution

European-Anatolian subspecies (Danilevsky 2021)

#### Stenurella nigra nigra

(Linnaeus, 1758)

2589E444-7176-506F-8DBF-715F94E574A4

##### Materials

- **Type status:** Other material. **Occurrence:** recordedBy: G. Georgiev leg. [GG]; sex: 1 male; **Location:** country: Bulgaria; locality: Ovtchartsi vill.; verbatimElevation: 900 m a.s.l.; **Event:** eventDate: 07-21-04

##### Distribution

European-Anatolian subspecies (Danilevsky 2021)

#### Stenurella bifasciata intermedia

Holzschuh, 2006

42E23E33-4877-5F87-AC17-9E53A4D02A51

##### Materials

- **Type status:** Other material. **Occurrence:** recordedBy: G. Georgiev leg. [GG]; **Location:** country: Bulgaria; locality: Ovtchartsi vill.; verbatimElevation: 900 m a.s.l.; **Event:** eventDate: 07-21-04

##### Distribution

Balkan endemic species (Danilevsky 2021)

#### Stenurella septempunctata septempunctata

(Fabricius, 1793)

4530C438-CC56-56BB-8E0C-F3A7E43E1B96

##### Materials

- **Type status:** Other material. **Occurrence:** recordedBy: G. Georgiev leg. [GG]; sex: 8 males, 4 females; **Location:** country: Bulgaria; locality: Harsovo vill.; verbatimElevation: 800 m a.s.l.; **Event:** eventDate: 07-20-04
- **Type status:** Other material. **Occurrence:** recordedBy: G. Georgiev leg. [GG]; sex: 1 male, 1 female; **Location:** country: Bulgaria; locality: Ovtchartsi vill.; verbatimElevation: 900 m a.s.l.; **Event:** eventDate: 07-21-04

##### Distribution

European subspecies (Danilevsky 2021)

#### Stenurella melanura melanura

(Linnaeus, 1758)

8B783E54-0F8A-5F74-9055-CFC39551107C

##### Materials

- **Type status:** Other material. **Occurrence:** recordedBy: G. Georgiev leg. [GG]; sex: 1 male; **Location:** country: Bulgaria; locality: Rila Monastery; verbatimElevation: 1400 m a.s.l.; **Event:** eventDate: 07-07-04
- **Type status:** Other material. **Occurrence:** recordedBy: G. Georgiev leg. [GG]; sex: 1 male, 1 female; **Location:** country: Bulgaria; locality: Harsovo vill.; verbatimElevation: 800 m a.s.l.; **Event:** eventDate: 07-20-04
- **Type status:** Other material. **Occurrence:** recordedBy: G. Georgiev leg. [GG]; sex: 1 female; **Location:** country: Bulgaria; locality: Parangalitsa; verbatimElevation: 1300 m a.s.l.; **Event:** eventDate: 07-20-04
- **Type status:** Other material. **Occurrence:** recordedBy: G. Georgiev leg. [GG]; sex: 1 male; **Location:** country: Bulgaria; locality: Treshtenik loc.; verbatimElevation: 1400 m a.s.l.; verbatimLatitude: 42.052222; verbatimLongitude: 23.668694

##### Distribution

Transpalaearctic subspecies (Danilevsky 2021)

#### Stictoleptura rubra rubra

(Linnaeus, 1758)

BD7AFCFF-3E16-5BED-BEF9-59C673E85288

##### Materials

- **Type status:** Other material. **Occurrence:** recordedBy: G. Georgiev leg. [GG]; sex: 1 female; **Location:** country: Bulgaria; locality: Ovtchartsi vill.; verbatimElevation: 900 m a.s.l.; **Event:** eventDate: 07-21-04

##### Distribution

Eurosiberian subspecies (Danilevsky 2021)

#### Paracorymbia maculicornis

(DeGeer, 1775)

5D5919A6-CCE2-5014-BBD7-85F67FD27145

##### Materials

- **Type status:** Other material. **Occurrence:** recordedBy: N. Simov leg. [GG]; sex: 1 male collected in tree traps on Pinus sylvestri; **Location:** country: Bulgaria; locality: Ravnite Mochuri loc. near Dobursko vill.; verbatimElevation: 1600 m a.s.l.; **Event:** eventDate: 7/6-8/30/2003
- **Type status:** Other material. **Occurrence:** recordedBy: G. Georgiev leg. [GG]; sex: 2 males, 1 female; **Location:** country: Bulgaria; locality: Rila Monastery; verbatimElevation: 1400 m a.s.l.; **Event:** eventDate: 07-07-04
- **Type status:** Other material. **Occurrence:** recordedBy: G. Georgiev leg. [GG]; sex: 3 males, 2 females; **Location:** country: Bulgaria; locality: Parangalitsa; verbatimElevation: 1300 m a.s.l.; **Event:** eventDate: 07-20-04

##### Distribution

European species (Danilevsky 2021)

#### Strangalia attenuata

(Linnaeus, 1758)

5B6F139F-43F2-591C-B29C-79A6995522FA

##### Materials

- **Type status:** Other material. **Occurrence:** recordedBy: G. Georgiev leg. [GG]; sex: 1 female; **Location:** country: Bulgaria; locality: Harsovo vill.; verbatimElevation: 800 m a.s.l.; **Event:** eventDate: 07-20-04
- **Type status:** Other material. **Occurrence:** recordedBy: G. Georgiev leg. [GG]; sex: 1 male; **Location:** country: Bulgaria; locality: Ovtchartsi vill.; verbatimElevation: 900 m a.s.l.; **Event:** eventDate: 07-21-04

##### Distribution

Transpalaearctic species (Danilevsky 2021)

#### Carilia virginea virginea

(Linnaeus, 1758)

EF7668D5-D32D-5A32-90B3-21BD64D92AD4

##### Materials

- **Type status:** Other material. **Occurrence:** recordedBy: G. Georgiev leg. [GG]; sex: 1 female; **Location:** country: Bulgaria; locality: Parangalitsa; verbatimElevation: 1300 m a.s.l.; **Event:** eventDate: 07-20-04
- **Type status:** Other material. **Occurrence:** recordedBy: G. Georgiev leg. [GG]; sex: 1 male; **Location:** country: Bulgaria; locality: Rila Monastery; verbatimElevation: 1400 m a.s.l.; **Event:** eventDate: 07-07-04

##### Distribution

Eurosiberian subspecies (Danilevsky 2021)

#### Cortodera humeralis humeralis

(Schaller, 1783)

6F525116-191D-55B1-92B2-F4F6761395AC

##### Materials

- **Type status:** Other material. **Occurrence:** recordedBy: N. Simov leg. [GG]; sex: 1 male, 1 female; **Location:** country: Bulgaria; locality: Above Dobarsko vill.; **Event:** eventDate: 06-02-03

##### Distribution

European-Anatolian subspecies (Danilevsky 2021)

#### Pachyta quadrimaculata

(Linnaeus, 1758)

8B5ED761-7334-50F4-812F-261DB38E8E9F

##### Materials

- **Type status:** Other material. **Occurrence:** recordedBy: G. Georgiev leg. [GG]; sex: 2 males, 1 female; **Location:** country: Bulgaria; locality: Parangalitsa; verbatimElevation: 1300 m a.s.l.; **Event:** eventDate: 07-20-04
- **Type status:** Other material. **Occurrence:** recordedBy: G. Georgiev leg. [GG]; sex: 2 males, 1 female; **Location:** country: Bulgaria; locality: Treshtenik loc.; verbatimElevation: 1400 m a.s.l.; verbatimLatitude: 42.052222; verbatimLongitude: 23.668694

##### Distribution

Transpalaearctic species (Danilevsky 2021)

#### Pidonia lurida

(Fabricius, 1793)

E001609C-2C1F-562A-9FF2-DEFEF6A5454B

##### Materials

- **Type status:** Other material. **Occurrence:** recordedBy: G. Georgiev leg. [GG]; sex: 1 female; **Location:** country: Bulgaria; locality: Rila Monastery; verbatimElevation: 1400 m a.s.l.; **Event:** eventDate: 07-07-04

##### Distribution

European species (Danilevsky 2021)

#### Rhagium bifasciatum

Fabricius, 1775

F65D681B-78E8-598D-B496-0D2E78608FF8

##### Materials

- **Type status:** Other material. **Occurrence:** recordedBy: P. Beron leg. [GG]; sex: 1 male, 1 female; **Location:** country: Bulgaria; locality: Malyovitsa, Chalat loc.; verbatimElevation: 2050 m a.s.l.; **Event:** eventDate: 06-07-64
- **Type status:** Other material. **Occurrence:** recordedBy: P. Beron leg. [GG]; sex: 1 male; **Location:** country: Bulgaria; locality: Maliovitsa; verbatimElevation: 2020 m a.s.l.; **Event:** eventDate: 11-01-70
- **Type status:** Other material. **Occurrence:** recordedBy: V. Radkova leg. [GG]; sex: 1 female; **Location:** country: Bulgaria; locality: Bistritsa vill.; verbatimElevation: 700 m a.s.l.; **Event:** eventDate: 06-06-81
- **Type status:** Other material. **Occurrence:** recordedBy: E. Andreeva leg. [GG]; sex: 1 male; **Location:** country: Bulgaria; locality: Blagoevgrad; verbatimElevation: 560 m a.s.l.; **Event:** eventDate: 06-28-82

##### Distribution

European-Iranoturanian species (Danilevsky 2021)

#### Xylosteus spinolae

Frivaldszky von Frivald, 1837

1F5DB1C0-237C-5E53-BAFD-5DC05D34B6F8

##### Materials

- **Type status:** Other material. **Occurrence:** recordedBy: G. Georgiev leg. [GG]; sex: 1 female; **Location:** country: Bulgaria; locality: Govedartsi vill.; verbatimElevation: 1200 m a.s.l.; **Event:** eventDate: 06-21-20

##### Distribution

Northeast Mediterranean species (Danilevsky 2021)

#### Cerambyx scopolii scopolii

Fuessly, 1775

1DD3FFAC-62DF-5241-B045-9E33342881A9

##### Materials

- **Type status:** Other material. **Occurrence:** recordedBy: V. Radkova leg. [GG]; sex: 1 male, 1 female; **Location:** country: Bulgaria; locality: Brashantsii vill.; **Event:** eventDate: 06-06-82

##### Distribution

European-Anatolian subspecies (Danilevsky 2021)

#### Clytus rhamni rhamni

Germar, 1817

6E200A65-6C1B-5E81-A8E9-6859FD8B0F64

##### Materials

- **Type status:** Other material. **Occurrence:** recordedBy: G. Georgiev leg. [GG]; sex: 3 males, 4 females; **Location:** country: Bulgaria; locality: Harsovo vill.; verbatimElevation: 800 m a.s.l.; **Event:** eventDate: 07-20-04

##### Distribution

Northeast Mediterranean subspecies (Danilevsky 2021)

#### Molorchus minor minor

(Linnaeus, 1758)

6BF96E9B-BDBD-5136-80F0-B467F3B0D94A

##### Materials

- **Type status:** Other material. **Occurrence:** recordedBy: G. Georgiev leg. [GG]; sex: 1 female; **Location:** country: Bulgaria; locality: Rila Monastery; verbatimElevation: 1400 m a.s.l.; **Event:** eventDate: 07-07-04

##### Distribution

Transpalaearctic subspecies (Danilevsky 2021)

#### Lamia textor

(Linnaeus, 1758)

BCB2FBE4-855F-5E67-A2AD-8540785430D7

##### Materials

- **Type status:** Other material. **Occurrence:** recordedBy: G. Georgiev leg. [GG]; sex: 1 female; **Location:** country: Bulgaria; locality: Blagoevgrad; verbatimElevation: 560 m a.s.l.; **Event:** eventDate: 05-14-90
- **Type status:** Other material. **Occurrence:** recordedBy: G. Georgiev leg. [GG]; sex: 1 female; **Location:** country: Bulgaria; locality: Harsovo vill.; verbatimElevation: 800 m a.s.l.; **Event:** eventDate: 07-20-04

##### Distribution

Transpalaearctic subspecies (Danilevsky 2021)

#### Monochamus sutor sutor

(Linnaeus, 1758)

D8FA95F7-EBA4-5F89-82D7-991B1B9CE016

##### Materials

- **Type status:** Other material. **Occurrence:** recordedBy: P. Mirchev leg. [GG]; sex: 1 male; **Location:** country: Bulgaria; locality: Iliina Riiver above Rila Monastery; **Event:** eventDate: 06-22-13

##### Distribution

West Eurosiberian subspecies Danilevsky 2021

#### Pogonocherus fasciculatus fasciculatus

DeGeer, 1775

B7A88EFE-1DA6-5D15-B2CA-B3AF1574FF2D

##### Materials

- **Type status:** Other material. **Occurrence:** recordedBy: N. Simov leg. [GG]; sex: 1 female; **Location:** country: Bulgaria; locality: Above Dobarsko vill.; **Event:** eventDate: 06-02-03

##### Distribution

Transpalaearctic subspecies (Danilevsky 2021)

## Analysis

In this study, two new taxa (*Stenurellanigranigra* and *Xylosteusspinolae*) were established for Rila Mt. New localities or additional information for 24 cerambycid taxa were also reported.

The total number of cerambycid taxa in Rila Mt. is 126 from six subfamilies: Prioninae (four taxa), Lepturinae (43 taxa), Necydalinae (two taxa), Spondylidinae (seven taxa), Cerambycinae (31 taxa) and Lamiinae (39 taxa) (Table 1).

Three taxa were previously reported under synonymous names: before revision of Danilevsky (2021), *Stenurellabifasciataintermedia* was reported as *Stenurellabifasciata*; recently described *Purpuricenuskaehlerirossicus* is distributed in Central and partly South Europe, including Bulgaria (Danilevsky 2021). Danilevsky (2019) mentioned that, in his collection, all materials of *Leiopusnebulosus* from Russia and adjacent countries, including Bulgaria, belong to recently described *Leiopuslinnei* (Wallin et al. 2009).

The established cerambycid taxa belong to 18 areographical categories separated in seven complexes (Table 2).

**Figure 1.**
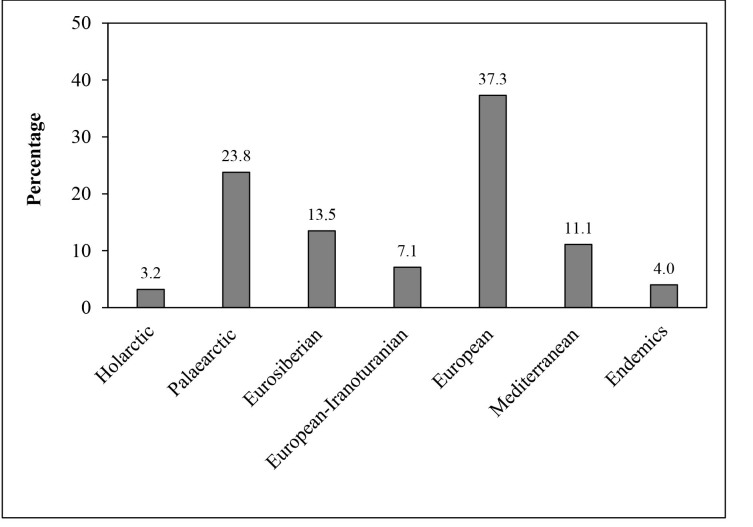
Arealographic complexes of cerambycides in Rila Mt.

The taxa from the European complex are dominant in Rila Mt. (37.3%), followed by those from Palaearctic (23.8%), Eurosiberian (13.5%) and Mediterranean (11.1%) complexes (Fig. 1).

## Discussion

The number of cerambycid taxa found in Rila Mt. (126 species and subspecies) is closest to that of Vitosha Mt. (122 taxa) (Topalov 2018). It is comparable to the number of cerambicides in other studied mountains in Bulgaria: Western Rhodopes (161 taxa) (Georgiev et al. 2006), West Balkan Range (107 taxa) (Georgiev 2011, Gradinarov and Petrova 2019) and Strandzha (154 taxa) (Georgiev et al. 2018).

In this study, the taxa of the European complex occupy a dominant position. They are connected with deciduous forests, which cover most of the mountainous territory of Rila. The second place is taken by the species and subspecies belonging to Palaearctic complex. These more euribiont taxa with broad areas of distribution normally are better presented in the high mountains, because of the harsh climatic conditions there. The third and fourth positions take taxa belonging to Eurosiberian and Mediterranean complexes. The high territories, mostly covered by coniferous trees and shrubs, are favourable for distribution of the Eurosiberian taxa. In the lower parts and along the rivers, conditions in Rila Mt. allow penetration of Mediterranean taxa. The refugial character of the region is underlined by the presence of five (4%) Balkan endemic cerambycids.

Similar aerographic characteristics of cerambycid fauna were established in Vitosha Mt. where European taxa (36.4%) take first place, followed by Palaearctic (20.6%), Eurosiberian (14.0%) and Mediterranean (12.4%) taxa (Topalov 2018). Vitosha with its elevation, oreographic patterns and vegetation is comparable to Rila Mt. Concerning the other two studied Mountains in Bulgaria - Strandzha (Georgiev et al. 2018) and Belasitsa (Georgiev et al. 2019), domination of European cerambycids was also established (33.1% and 38.2%, respectively), but the Mediterranean taxa take a greater share in both mountains (27.3% and 19.1%, respectively). In addition, European-Iranoturanian taxa are mostly represented in Strandzha Mt. (13.6%) compared to other mountains (7.2-11.1%). The level of Balkan and Bulgarian endemics is higher in Belasitsa Mt. – nine taxa (8.2%), followed by Rila Mt. – five taxa (4.0%), Strandzha Mt. – five taxa (3.3%) and Vitosha Mt. – two taxa (1.7%). Evidently, the conditions in Belasitsa Mt. and, especially, the distribution of relict forests of *Castaneasativa*, are the most suitable for occurrence of endemics there.

In conclusion, it should be noted that the finding of 126 taxa (approximately 45% of longhorn beetles in Bulgaria) indicates that this taxonomic group is not yet well-studied and about 50 species and subspecies are expected to be found in future investigations in Rila Mt.

## Supplementary Material

XML Treatment for Prionus coriarius

XML Treatment for Alosterna tabacicolor tabacicolor

XML Treatment for Anastrangalia dubia dubia

XML Treatment for Anastrangalia sanguinolenta

XML Treatment for Leptura quadrifasciata quadrifasciata

XML Treatment for Judolia cerambyciformis

XML Treatment for Rutpela maculata maculata

XML Treatment for Stenurella nigra nigra

XML Treatment for Stenurella bifasciata intermedia

XML Treatment for Stenurella septempunctata septempunctata

XML Treatment for Stenurella melanura melanura

XML Treatment for Stictoleptura rubra rubra

XML Treatment for Paracorymbia maculicornis

XML Treatment for Strangalia attenuata

XML Treatment for Carilia virginea virginea

XML Treatment for Cortodera humeralis humeralis

XML Treatment for Pachyta quadrimaculata

XML Treatment for Pidonia lurida

XML Treatment for Rhagium bifasciatum

XML Treatment for Xylosteus spinolae

XML Treatment for Cerambyx scopolii scopolii

XML Treatment for Clytus rhamni rhamni

XML Treatment for Molorchus minor minor

XML Treatment for Lamia textor

XML Treatment for Monochamus sutor sutor

XML Treatment for Pogonocherus fasciculatus fasciculatus

## Figures and Tables

**Table 1.** Localities and chorotypes of longhorn beetles (Coleoptera: Cerambycidae) in Rila Mt.

**N**	**Taxon**	**Locality**	**References**	**Chorotype**
	**Subfamily Prioninae Latreille, 1802**			
1	*Tragosomadepsarium* (Linnaeus, 1767)	Blagoevgrad	Kantardjiewa-Minkova 1932	Eurosiberian
2	*Mesoprionusbesikanus* (Fairmaire, 1855)	Rila Mt.	Kantardjiewa-Minkova 1932, Angelov 1995	East Mediterranean
3	*Prionuscoriarius* (Linnaeus, 1758)	Yundola Borovets, Kostenets Rila Mt. Bistritsa Brashantsi vill.	Kantardjiewa-Minkova 1932, Samuelian 1998, Kantardjiewa-Minkova 1932, Angelov 1995, Georgiev 2020, **New record**	West Palaearctic
4	*Ergatesfaber* (Linnaeus, 1760)	Rila Monastery Rila Mt.	Kantardjiewa-Minkova 1932, Angelov 1995	West Palaearctic
	**Subfamily Lepturinae Latreille, 1802**			
5	*Alosternatabacicolortabacicolor* (DeGeer, 1775)	Rila Mt. Parangalitsa	Kantardjiewa-Minkova 1932, **New record**	West Eurosiberian
6	*Anastrangaliadubiadubia* (Scopoli, 1763)	Rila Monastery Borovets Rila Mt. Parangalitsa, Treshtenik	Kantardjiewa-Minkova 1932, Minkova 1957, Minkova 1957, Ganev 1985, Gradinarov et al. 2020, Angelov 1995, **New records**	Euromediterranean
7	*Anastrangaliasanguinolenta* (Linnaeus, 1760)	Kostenets Rila Mt. Gorno Osenovo Borovets Parangalitsa, Treshtenik	Heyrovský 1931, Kantardjiewa-Minkova 1932, Angelov 1995, Georgiev 2020, Gradinarov et al. 2020, **New records**	West Eurosiberian
8	*Anoploderarufipesrufipes* (Schaller, 1783)	Rila Mt.	Heyrovský 1931, Angelov 1995	European
9	*Anoploderasexguttata* (Fabricius, 1775)	Rila Mt.	Angelov 1995	Euromediterranean
10	*Grammopteraabdominalis* (Stephens, 1831)	Rila Mt.	Heyrovský 1931, Kantardjiewa-Minkova 1932, Angelov 1995	European-Iranian
11	*Grammopteraustulataustulata* (Schaller, 1783)	Rila Mt.	Heyrovský 1931, Kantardjiewa-Minkova 1932, Angelov 1995	European-Anatolian
12	*Lepturaaurulenta* Fabricius, 1793	Yundola	Ganev 1986	Euromediterranean
13	*Lepturaquadrifasciataquadrifasciata* Linnaeus, 1758	Raduil Rila Mt. Belmeken Yundola Parangalitsa	Heyrovský 1931, Kantardjiewa-Minkova 1932, Minkova 1957, Samuelian 1998, **New records**	Transpalaearctic
14	*Lepturoboscavirens* (Linnaeus, 1758)	Rila Mt.	Angelov 1995	Transpalaearctic
15	*Judoliacerambyciformis* (Schrank, 1781)	Rila Mt. Borovets Yundola, Musala hut Kostenets Rila Monastery Harsovo, Parangalitsa, Ovtchartsi, Treshtenik	Heyrovský 1931, Kantardjiewa-Minkova 1932, Minkova 1957, Georgiev 2020, Angelov 1967, Minkova 1957, Ganev 1985, Ganev 1985, Gradinarov et al. 2020, **New records**	European
16	*Judoliaerraticus* (Dalman, 1817)	Kostenets Yundola, Samokov Blagoevgrad Rila Monastery	Heyrovský 1931, Minkova 1957, Angelov 1967, Georgiev 2020, Gradinarov et al. 2020	European-Iranian
17	*Pseudovadonialividalivida* (Fabricius, 1777)	Borovets	Kantardjiewa-Minkova 1932	European
18	*Rutpelamaculatamaculata* (Poda von Neuhaus, 1761)	Borovets, Kostenets Belmeken Harsovo Govedartsi Rila Monastery Ovchartsi, Treshtenik	Kantardjiewa-Minkova 1932, Minkova 1957, Rapuzzi and Georgiev 2007, Georgiev 2020, Gradinarov and Petrova 2019, **New records**	European-Anatolian
19	*Stenurellanigranigra* (Linnaeus, 1758)	Ovchartsi	**New record**	European-Anatolian
20	*Stenurellabifasciataintermedia* Holzschuh, 2006	Blagoevgrad Borovets Ovtchartsi	Georgiev 2020, Gradinarov et al. 2020, **New record**	Balkan endemic
21	*Stenurellaseptempunctataseptempunctata* (Fabricius, 1793)	Borovets Rila Monastery Harsovo, Ovtchartsi	Kantardjiewa-Minkova 1932, Gradinarov et al. 2020, **New records**	European
22	*Stenurellamelanuramelanura* (Linnaeus, 1758)	Borovets Rila Monastery Blagoevgrad, Predela Harsovo, Parangalitsa, Treshtenik	Heyrovský 1931, Angelov 1967, Georgiev 2020, **New records**	Transpalaearctic
23	*Stictolepturarubrarubra* (Linnaeus, 1758)	Borovets Raduil, Samokov, Kostenets Rila Monastery Yundola Yakoruda Ovtchartsi	Heyrovský 1931, Kantardjiewa-Minkova 1932, Gradinarov et al. 2020, Kantardjiewa-Minkova 1932, Angelov 1967, Samuelian 1998, Georgiev 2020, **New record**	Eurosiberian
24	*Paracorymbiamaculicornis* (DeGeer, 1775)	Borovets Rila Mt. Dobarsko, Rila Monastery, Parangalitsa	Kantardjiewa-Minkova 1932, Gradinarov et al. 2020, Angelov 1995, **New records**	European
25	*Stictolepturascutellatascutellata* (Fabricius, 1781)	Belmeken	Minkova 1957	European
26	*Paracorymbiafulva* (DeGeer, 1775)	Kostenets Rila Monastery Harsovo, Ovchartsi	Heyrovský 1931, Kantardjiewa-Minkova 1932, Kantardjiewa-Minkova 1932, Rapuzzi and Georgiev 2007	European-Anatolian
27	*Stictolepturaerythroptera* (Hagenbach, 1822)	Borovets	Heyrovský 1931, Kantardjiewa-Minkova 1932	European-Iranian
28	*Strangaliaattenuata* (Linnaeus, 1758)	Rila Mt. Belmeken Borovets Harsovo, Ovtchartsi	Kantardjiewa-Minkova 1932, Minkova 1957, Ganev 1985, **New records**	Transpalaearctic
29	*Vadoniaunipunctataunipunctata* (Fabricius, 1787)	Rila Monastery	Kantardjiewa-Minkova 1932	European-Anatolian
30	*Oxymiruscursor* (Linnaeus, 1758)	Rila Mt. Borovets Rila Monastery, Kostenets	Heyrovský 1931, Angelov 1995, Kantardjiewa-Minkova 1932, Minkova 1957, Ganev 1985, Kantardjiewa-Minkova 1932	West Eurosiberian
31	*Cariliavirgineavirginea* (Linnaeus, 1758)	Borovets Soleno Dere Rila Monastery Yundola Eleshnitsa River Parangalitsa	Kantardjiewa-Minkova 1932, Minkova 1957, Kantardjiewa-Minkova 1934, Angelov 1967, Samuelian 1998, Georgiev 2020, **New record**	West Eurosiberian
32	*Cortoderahumeralishumeralis* (Schaller, 1783)	Kostenets, Rila Monastery Dobarsko	Kantardjiewa-Minkova 1932, Minkova 1957, **New record**	European-Anatolian
33	*Cortoderaflavimanaflavimana* (Waltl, 1838)	Yundola	Samuelian 1998	European-Anatolian
34	*Dinopteracollaris* (Linnaeus, 1758)	Rila Mt. Rila Monastery	Kantardjiewa-Minkova 1932, Gradinarov et al. 2020	Eurosiberian
35	*Acmaeopsseptentrionis* (C. G. Thomson, 1866)	Parangalitsa Borovets, Dupnitsa	Kantardjiewa-Minkova 1932, Minkova 1957, Angelov 1995, Minkova 1957, Angelov 1995, Angelov 1995	Transpalaearctic
36	*Evodinellusclathratus* (Fabricius, 1793)	Rila Mt.	Angelov 1995	European
37	*Acmaeopspratensis* (Laicharting, 1784)	Borovets Rila Mt.	Kantardjiewa-Minkova 1932, Angelov 1995	Transholarctic
38	*Pachytalamed* (Linnaeus, 1758)	Borovets Rila Mt.	Kantardjiewa-Minkova 1932, Angelov 1995	Transpalaearctic
39	*Pachytaquadrimaculata* (Linnaeus, 1758)	Borovets Kostenets, Belmeken Rila Mt. Parangalitsa, Treshtenik	Kantardjiewa-Minkova 1932, Ganev 1985, Gradinarov et al. 2020, Kantardjiewa-Minkova 1932, Angelov 1995, **New records**	Transpalaearctic
40	*Pidonialurida* (Fabricius, 1793)	Samokov Borovets Rila Monastery	Heyrovský 1931, Kantardjiewa-Minkova 1932, **New record**	European
41	*Rhagiumbifasciatum* Fabricius, 1775	Rila Mt. Borovets Sitnyakovo Kostenets Rila Monastery Yundola Malyovitsa Bistritsa, Blagoevgrad	Heyrovský 1931, Kantardjiewa-Minkova 1932, Angelov 1967, Kantardjiewa-Minkova 1932, Kantardjiewa-Minkova 1932, Minkova 1957, Ganev 1984, Ganev 1985, Samuelian 1998, Georgiev 2020, **New records**	European-Iranian
42	*Rhagiummordax* (DeGeer, 1775)	Rila Mt. Borovets, Dolna Banya Kostenets, Parangalitsa	Heyrovský 1931, Kantardjiewa-Minkova 1932, Kantardjiewa-Minkova 1932, Ganev 1986, Doychev et al. 2017	Eurosiberian
43	*Rhagiumsycophanta* (Schrank, 1781)	Yundola	Samuelian 1998	West Eurosiberian
44	*Rhagiuminquisitorinquisitor* (Linnaeus, 1758)	Parangalitsa Kostenets Borovets, Samokov Yundola, Bodrost Chalet	Drenski 1933, Kantardjiewa-Minkova 1932, Ganev 1984, Kantardjiewa-Minkova 1932, Samuelian 1998, Doychev et al. 2017, Gradinarov et al. 2020	Eurosiberian
45	*Stenocorusmeridianus* (Linnaeus, 1758)	Borovets Rila Mt.	Ganev 1985, Angelov 1995	Eurosiberian
46	*Xylosteusbartoni* Obenberger & Mařan, 1933	Borovets, Parangalitsa	Minkova 1957, Angelov 1995, Georgiev and Simov 2006	Balkan endemic
47	*Xylosteusspinolae* Frivaldszky von Frivald, 1837	Govedartsi	**New record**	Northeast Mediterranean
	**Subfamily Necydalinae Latreille, 1825**			
48	*Necydalismajor* Linnaeus, 1758	Borovets	Kantardjiewa-Minkova 1932	Transpalaearctic
49	*Necydalisulmi* Chevrolat, 1838	Borovets	Angelov 1995	European-Anatolian
	**Subfamily Spondylidinae Audinet-Serville, 1832**			
50	*Alocerusmoesiacus* (Frivaldszky von Frivald, 1837)	Yundola	Samuelian 1998	Transmediterranean
51	*Archopalusrusticusrusticus* (Linnaeus, 1758)	Borovets, Blagoevgrad Predela	Kantardjiewa-Minkova 1932, Georgiev 2020	Transpalaearctic
52	*Asemumstriatum* (Linnaeus, 1758)	Kostenets	Minkova 1957	Transholarctic
53	*Tetropiumcastaneum* (Linnaeus, 1758)	Parangalitsa Borovets Yundola Deno Peak	Drenski 1933, Kantardjiewa-Minkova 1932, Samuelian 1998, Gradinarov et al. 2020	Transpalaearctic
54	*Tetropiumfuscumfuscum* (Fabricius, 1787)	Rila Mt. Yundola Parangalitsa	Kantardjiewa-Minkova 1932, Angelov 1995 Ganev 1986, Samuelian 1998, Angelov 1995	Transholarctic
55	*Saphanuspiceusganglbaueri* Brancsik, 1886	Borovets	Heyrovský 1931, Kantardjiewa-Minkova 1932, Angelov 1995	European
56	*Spondylisbuprestoides* (Linnaeus, 1758)	Rila Mt. Panichishte Govedartsi Yundola	Kantardjiewa-Minkova 1932, Ganev 1984, Ganev 1986, Samuelian 1998	Transpalaearctic
	**Subfamily Cerambycinae Latreille, 1802**			
57	*Anaglyptusmysticus* (Linnaeus, 1758)	Rila Monastery	Gradinarov et al. 2020	European-Anatolian
58	*Aromiamoschatamoschata* (Linnaeus, 1758)	Borovo	Kantardjiewa-Minkova 1932	Eurosiberian
59	*Callidiumviolaceum* (Linnaeus, 1758)	Borovets	Kantardjiewa-Minkova 1932	Transpalaearctic
60	*Callidiumaeneumaeneum* (De Deer, 1775)	Rila Monastery	Kantardjiewa-Minkova 1932, Doychev et al. 2017	Transpalaearctic
61	*Callidiumcoriaceum* Paykull, 1800	Yundola	Doychev and Bencheva 2008	Transpalaearctic
62	*Lioderinalinearis* (Hampe, 1871)	Blagoevgrad	Ganev 1984	Northeast Mediterranean
63	*Pyrrhidiumsanguineum* (Linnaeus, 1758)	Yundola	Samuelian 1998	Euromediterranean
64	*Ropalopusclavipes* (Fabricius, 1775)	Rila Monastery Yundola	Kantardjiewa-Minkova 1932, Samuelian 1998	European-Iranian
65	*Ropalopusungaricusinsubricus* (Germar, 1823)	Rila Monastery	Ganev 1986	European
66	*Cerambyxmiles* Bonelli, 1812	Kostenets	Kantardjiewa-Minkova 1932	European-Anatolian
67	*Cerambyxnodulosusnodulosus* Germar, 1817	Rila Monastery	Ganev 1985	Pontomediterranean
68	*Cerambyxscopoliiscopolii* Fuessly, 1775	Rila Mt. Brashantsii	Heyrovský 1931, **New record**	European-Anatolian
69	*Chlorophorusherbstii* (Brahm, 1790)	Rila Mt. Kostenets	Kantardjiewa-Minkova 1932, Ganev 1986	Eurosiberian
70	*Clytusarietisarietis* (Linnaeus, 1758)	Rila Mt.	Heyrovský 1931, Kantardjiewa-Minkova 1932	European-Anatolian
71	*Clytuslama* Mulsant, 1847	Kostenets	Kantardjiewa-Minkova 1932	European
72	*Clytusrhamnirhamni* Germar, 1817	Kostenets Harsovo	Kantardjiewa-Minkova 1932, **New record**	Northeast Mediterranean
73	*Plagionotusarcuatusarcuatus* (Linnaeus, 1758)	Rila Mt. Yundola Razlog	Heyrovský 1931, Samuelian 1998, Georgiev 2020	Euromediterranean
74	*Plagionotusdetritusdetritus* (Linnaeus, 1758)	Kostenets Borovets	Kantardjiewa-Minkova 1932, Kantardjiewa-Minkova 1934, Kantardjiewa-Minkova 1932	European-Anatolian
75	*Xylotrechusrusticus* (Linnaeus, 1758)	Rila Mt.	Kantardjiewa-Minkova 1932	Transpalaearctic
76	*Xylotrechusarvicolaarvicola* (Olivier, 1795)	Predela	Georgiev 2020	Euromediterranean
77	*Rosalia alpina* (Linnaeus, 1758)	Borovets, Kostenets	Kantardjiewa-Minkova 1932	European-Anatolian
78	*Stromatiumauratum* (Böber, 1793)	Blagoevgrad	Georgiev 2020	Transmediterranean
79	*Molorchusminorminor* (Linnaeus, 1758)	Parangalitsa Borovets Rila Mt. Rila Monastery	Drenski 1933, Kantardjiewa-Minkova 1932, Angelov 1995, **New record**	Transpalaearctic
80	*Molorchusumbellatarumumbellatarum* (Schreber, 1759)	Rila Mt.	Heyrovský 1931, Kantardjiewa-Minkova 1932, Angelov 1995	European-Iranoturanian
81	*Obriumbrunneum* (Fabricius, 1793)	Rila Monastery	Georgiev et al. 2005	European-Anatolian
82	*Purpuricenusbudensis* (Götz, 1783)	Yundola	Samuelian 1998	West Palaearctic
83	*Purpuricenuskaehlerirossicus* Danilevsky, 2019	Raduil Rila	Kantardjiewa-Minkova 1932, Ganev 1985	East European
84	*Callimusangulatus*angulatus (Schrank, 1789)	Rila Mt.	Heyrovský 1931, Kantardjiewa-Minkova 1932	Euromediterranean
85	*Callimusfemoratus* (Germar, 1824)	Yundola	Samuelian 1998	East European-Iranian
86	*Stenopterusflavicornis* Küster, 1846	Kostenets	Kantardjiewa-Minkova 1932	Northeast Mediterranean
87	*Stenopterusrufusrufus* (Linnaeus, 1767)	Harsovo, Parangalitsa	Rapuzzi and Georgiev 2007	European
	**Subfamily Lamiinae Latreille, 1825**			
88	*Acanthocinusaedilis* (Linnaeus, 1758)	Borovets Samokov	Kantardjiewa-Minkova 1934, Ganev 1985	Transpalaearctic
89	*Acanthocinusgriseus* (Fabricius, 1793)	Samokov	Hubenov et al. 2001, Doychev et al. 2017	Transpalaearctic
90	*Leiopuslinnei* Wallin, Nylander & Kvamme, 2009	Rila Mt. Belmeken	Heyrovský 1931, Kantardjiewa-Minkova 1934	European
91	*Agapanthiacynaraecynarae* (Germar, 1817)	Dupnitsa Borovets	Kantardjiewa-Minkova 1934, Ganev 1985	North Mediterranean
92	*Agapanthiadahlidahli* (C. F. W. Richter, 1820)	Borovets	Kantardjiewa-Minkova 1934, Ganev 1985	European
93	*Agapanthiavillosoviridescens* (De Geer, 1775)	Rila Mt. Rila Monastery Borovets	Heyrovský 1931, Kantardjiewa-Minkova 1934, Angelov 1967, Ganev 1985	Eurosiberian
94	*Agapanthiaviolacea* (Fabricius, 1775)	Rila Mt. Blagoevgrad, Bistritsa, Rila Monastery	Heyrovský 1931, Georgiev 2020	European-Anatolian
95	*Agapanthiakirbyikirbyi* (Gyllenhal, 1817)	Borovets Parangalitsa	Heyrovský 1931, Kantardjiewa-Minkova 1934, Georgiev 2020	European-Iranian
96	*Anaesthetistestaceatestacea* (Fabricius, 1781)	Predela	Georgiev 2020	European-Anatolian
97	*Stenideageneigenei* (Aragona, 1830)	Kostenets	Angelov 1967	North Mediterranean
98	*Dorcadionaethiopsaethiops* (Scopoli, 1763)	Rila Mt. Borovets Samokov	Kantardjiewa-Minkova 1934, Minkova 1961, Ganev 1986	Northeast Mediterranean
99	*Dorcadionfulvumerythropterum* Fischer von Waldheim, 1823	Blagoevgrad Yundola	Ganev 1985, Samuelian 1998	East European
100	*Dorcadionaxillare* Küster, 1847	Rila Mt.	Kantardjiewa-Minkova 1934, Minkova 1961	Balkan endemic
101	*Dorcadionsturmii* Frivaldszky von Frivald, 1837	Kostenets	Minkova 1961	Balkan endemic
102	*Dorcadiontauricumtauricum* Waltl, 1838	Rila Mt.	Heyrovský 1931	East European
103	*Dorcadionpedestrepedestre* (Poda von Neuhaus, 1761)	Yundola	Samuelian 1998	Northeast Mediterranean
104	*Neodorcadionbilineatum* (Germar, 1823)	Kostenets	Heyrovský 1931	Northeast Mediterranean
105	*Lamiatextor* (Linnaeus, 1758)	Kostenets Blagoevgrad, Harsovo	Kantardjiewa-Minkova 1934, **New records**	Transpalaearctic
106	*Morimusasperfunereus* Mulsant, 1862	Rila Mt. Yundola	Heyrovský 1931, Samuelian 1998	Northeast Mediterranean
107	*Mesosacurculionoides* (Linnaeus, 1760)	Rila Mt. Blagoevgrad	Heyrovský 1931, Kantardjiewa-Minkova 1934, Rapuzzi and Georgiev 2007	European-Iranian
108	*Monochamusgalloprovincialispistor* (Germar, 1818)	Parangalitsa	Doychev et al. 2017	West Eurosiberian
109	*Monochamussartor* (Fabricius, 1787)	Parangalitsa Kostenets	Drenski 1933, Ganev 1985	European
110	*Monochamussutorsutor* (Linnaeus, 1758)	Rila Mt. Yundola Rila Monastery, Borovets Iliina River	Heyrovský 1931, Samuelian 1998, Gradinarov et al. 2020, **New record**	West Eurosiberian
111	*Obereaerythrocephalaerythrocephala* (Schrank, 1776)	Rila Monastery, Borovets	Gradinarov et al. 2020	West Palaearctic
112	*Phytoeciaaffinisaffinis* (Harrer, 1784)	Rila Mt. Rila Monastery	Heyrovský 1931, Kantardjiewa-Minkova 1934, Bringmann 1998, Gradinarov et al. 2020	European-Anatolian
113	*Phytoeciacoerulescenscoerulescens* (Scopoli, 1763)	Rila Mt.	Heyrovský 1931	West Palaearctic
114	*Phytoeciacylindrica* (Linnaeus, 1758)	Rila Mt. Kostenets	Heyrovský 1931, Kantardjiewa-Minkova 1934	Transpalaearctic
115	*Phytoeciageniculataorientalis* Kraatz, 1871	Borovets	Migliaccio et al. 2007	Balkan endemic
116	*Phytoeciaicterica* (Schaller, 1783)	Samokov	Heyrovský 1931, Kantardjiewa-Minkova 1934	West Palaearctic
117	*Phytoecianigricornis* (Fabricius, 1782)	Rila Mt.	Heyrovský 1931, Kantardjiewa-Minkova 1934	Eurosiberian
118	*Phytoeciavirgula*virgula (Charpentier, 1825)	Rila Mt.	Heyrovský 1931, Kantardjiewa-Minkova 1934	Transpalaearctic
119	*Pogonocherusfasciculatusfasciculatus* DeGeer, 1775	Rila Mt. Borovets Yundola, Belmeken Dobarsko	Tschorbadjiew 1927, Kantardjiewa-Minkova 1934, Doychev et al. 2017, **New record**	Transpalaearctic
120	*Pogonocherushispidulus* (Piller & Mitterpacher, 1783)	Rila Mt. Yakoruda	Heyrovský 1931, Kantardjiewa-Minkova 1934, Bringmann and Dring 2001	Euromediterranean
121	*Saperdapopulnea* (Linnaeus, 1758)	Rila Mt. Kostenets Samokov	Heyrovský 1931, Kantardjiewa-Minkova 1934, Georgiev et al. 2004	Transholarctic
122	*Saperdaoctopunctata* (Scopoli, 1772)	Kostenets	Kantardjiewa-Minkova 1934, Ganev 1986	European-Anatolian
123	*Saperdascalarisscalaris* (Linnaeus, 1758)	Rila Mt. Kostenets	Heyrovský 1931, Kantardjiewa-Minkova 1934, Ganev 1986	Euromediterranean
124	*Saperdacarcharias* (Linnaeus, 1758)	Kostenets	Ganev 1985	Transpalaearctic
125	*Stenostolaferreaferrea* (Schrank, 1776)	Kostenets	Kantardjiewa-Minkova 1934	European-Anatolian
126	*Tetropspraeustuspraeustus* (Linnaeus, 1758)	Rila Mt.	Heyrovský 1931	Transpalaearctic

**Table 2.** Areogeographic characterisation of cerambycids in Rila Mt.

**Areographic categories and complexes**	**Number**	**Percentage**
**Holarctic complex**	**4**	**3.2**
Transholarctic	4	3.2
**Palaearctic complex**	**30**	**23.8**
Transpalaearctic	24	19.0
West Palaearctic	6	4.8
**Eurosiberian complex**	**17**	**13.5**
Eurosiberian	10	7.9
West Eurosiberian	7	5.6
**European-Iranoturanian complex**	**9**	**7.1**
European-Iranoturanian	1	0.8
European-Iranian	7	5.5
East European-Iranian	1	0.8
**European complex**	**47**	**37.3**
Euromediterranean	9	7.1
European-Anatolian	20	15.9
European	15	11.9
East European	3	2.4
**Mediterranean complex**	**14**	**11.1**
Transmediterranean	2	1.6
North Mediterranean	2	1.6
East Mediterranean	1	0.8
Northeast Mediterranean	8	6.3
Pontomediterranean	1	0.8
**Balkan endemic complex**	**5**	**4.0**
Balkan endemics	5	4.0
**Total**	**126**	**100.0**
